# Single‐Cell Transcriptome Analysis Uncovers Intratumoral Heterogeneity and Underlying Mechanisms for Drug Resistance in Hepatobiliary Tumor Organoids

**DOI:** 10.1002/advs.202003897

**Published:** 2021-03-23

**Authors:** Yan Zhao, Zhi‐Xuan Li, Yan‐Jing Zhu, Jing Fu, Xiao‐Fang Zhao, Ya‐Ni Zhang, Shan Wang, Jian‐Min Wu, Kai‐Ting Wang, Rui Wu, Cheng‐Jun Sui, Si‐Yun Shen, Xuan Wu, Hong‐Yang Wang, Dong Gao, Lei Chen

**Affiliations:** ^1^ School of Life Sciences and Institute of Metabolism and Integrative Biology Fudan University Shanghai 200438 China; ^2^ National Center for Liver Cancer Shanghai 200441 China; ^3^ The International Cooperation Laboratory on Signal Transduction Eastern Hepatobiliary Surgery Hospital Second Military Medical University Shanghai 200438 China; ^4^ Fudan University Shanghai Cancer Center Department of Oncology Shanghai Medical College Fudan University Shanghai 200032 China; ^5^ Institute of Metabolism and Integrative Biology Fudan University Shanghai 200438 China; ^6^ Eastern Hepatobiliary Surgery Hospital Second Military Medical University Shanghai 200438 China; ^7^ Department of Laboratory Medicine The Tenth People's Hospital of Shanghai Tongji University Shanghai 200072 China; ^8^ The State Key Laboratory of Cell Biology Shanghai Key Laboratory of Molecular Andrology CAS Center for Excellence in Molecular Cell Science Shanghai Institute of Biochemistry and Cell Biology University of Chinese Academy of Sciences Chinese Academy of Sciences Shanghai 200031 China; ^9^ Institute for Stem Cell and Regeneration Chinese Academy of Sciences Beijing 100101 China

**Keywords:** drug resistance, hepatobiliary tumor organoid, single‐cell analysis, tumor ecosystem, tumor heterogeneity

## Abstract

Molecular heterogeneity of hepatobiliary tumor including intertumoral and intratumoral disparity always leads to drug resistance. Here, seven hepatobiliary tumor organoids are generated to explore heterogeneity and evolution via single‐cell RNA sequencing. HCC272 with high status of epithelia‐mesenchymal transition proves broad‐spectrum drug resistance. By examining the expression pattern of cancer stem cells markers (e.g., PROM1, CD44, and EPCAM), it is found that CD44 positive population may render drug resistance in HCC272. UMAP and pseudo‐time analysis identify the intratumoral heterogeneity and distinct evolutionary trajectories, of which catenin beta‐1 (CTNNB1), glyceraldehyde‐3‐phosphate dehydrogenase (GAPDH), and nuclear paraspeckle assembly transcript 1 (NEAT1) advantage expression clusters are commonly shared across hepatobiliary organoids. CellphoneDB analysis further implies that metabolism advantage organoids with enrichment of hypoxia signal upregulate NEAT1 expression in CD44 subgroup and mediate drug resistance that relies on Jak‐STAT pathway. Moreover, metabolism advantage clusters shared in several organoids have similar characteristic genes (GAPDH, NDRG1 (N‐Myc downstream regulated 1), ALDOA, and CA9). The combination of GAPDH and NDRG1 is an independent risk factor and predictor for patient survival. This study delineates heterogeneity of hepatobiliary tumor organoids and proposes that the collaboration of intratumoral heterogenic subpopulations renders malignant phenotypes and drug resistance.

## Graphical abstract

The existence of inter‐ and intratumoral heterogeneity is the main cause for tumor drug resistance. Thus, extensive understanding of the underlying mechanism is necessary for developing potential strategy. This study here, for the first time, provides the new understanding for the role of tumor ecosystem involving cell expansion and drug response by applying scRNA‐seq method with tumor organoids.

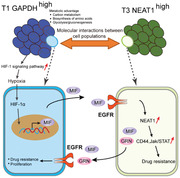

## 1 Introduction

Extensive genetic and phenotypic variation exist not only among tumors from different patients (intertumoral heterogeneity) but also within individual tumors (inratumoral heterogeneity).^[^ ^1^ ^]^ Tumor heterogeneity renders diversity of cancer signaling pathways and drives phenotypic variation, which poses a significant challenge to personalized cancer medicine.^[^ ^2^ ^]^ Transcriptomic diversity and cancer stem cells (CSCs) plasticity^[^ ^3^ ^]^ are prominent causes of heterogeneity in cancer, generating diverse cell populations that can be subjected to selection in the given microenvironment. The development of single‐cell level high‐throughput sequencing technology has accelerated the understanding of driver gene mutations, aberrant regulatory programs, and molecular subtypes for human tumors.^[^ ^4^ ^]^ However, most of existing studies relied on profiling technologies that measure tumors in bulk, which is insufficient to globally explore and explain the heterogeneity and evolution.

Recent advances in single‐cell technology provide an avenue to explore the characteristics of the genome, transcriptome, and epigenome at the single‐cell level.^[^ ^5^ ^]^ In the past several years, single‐cell RNA sequencing (scRNA‐seq) has been applied in landscape construction of many parenchymal and nonparenchymal cells to redefine cell‐type compositions and discover new subsets in physiological and pathological conditions. In addition, several studies revealed new insights into evolution and diversity in many human solid tumors, including intracranial tumor,^[^ ^6^ ^]^ head and neck cancer,^[^ ^7^ ^]^ breast cancer,^[^ ^8^ ^]^ and lung cancer via scRNA‐seq. Currently, researches on hepatobiliary system were reported successively, however, the intrinsic between heterogeneity and drug response in hepatobiliary tumor is still unclear.

Precision oncology seeks to develop more physiological human cancer models. The emerging organoid technology allows the in vitro long‐term culture of patient‐derived cancer cells which faithfully recapitulates the in vivo phenotype.^[^ ^7^, ^8^ ^]^ Organoids are derived from pluripotent stem cells or isolated organ progenitors that differentiate to form an organ‐like tissue exhibiting multiple cell types.^[^ ^7^, ^8^ ^]^ Recently, organoids have been applied to model various cancers, including prostate,^[^ ^9^ ^]^ pancreatic,^[^ ^10^ ^]^ breast,^[^ ^11^ ^]^ liver,^[^ ^12^ ^]^ bladder,^[^ ^13^ ^]^ and gastrointestinal cancers,^[^ ^14^ ^]^ and facilitate extensive delineation of the phenotypic and molecular heterogeneity within tumors. It can preserve the genetic alterations and gene expression of the original tumor,^[^ ^14^, ^15^ ^]^ possess metastatic potential in vivo,^[^ ^15^ ^]^ and thereby mirror features of the original tumor, including intratumoral heterogeneity. Tumor‐derived^[^ ^16^ ^]^ organoid has become a powerful tool for tumor biology, stem cell biology, and drug‐discovery researches.

Owing to tremendous heterogeneity, it is a big challenge to establish a research model that can faithfully recapitulate the in vivo phenotype and further investigate the heterogeneity and drug resistance in hepatobiliary tumors. Here, we established patient‐derived hepatobiliary tumor organoids from seven patients and employed scRNA‐seq to dissect intertumoral and intratumoral heterogeneity, which revealed the inherent variable in their expression of transcriptional programs related to cell cycle, hypoxia, and epithelial status. In addition, we identified that CSCs heterogeneity may contribute to a molecular and biological diversity in tumor ecosystems and consequently drug responses. Further, we demonstrated unique distinctive metabolic circuitry in resistant subpopulations, which may hold the key of distinct molecular signatures and drug resistance. These data revealed new insight into tumoral heterogeneity of hepatobiliary tumor organoids and associated critical subpopulations in regulating tumor drug resistance.

## 2 Results

### 2.1 Establishment of Patient‐Derived Hepatobiliary Tumor Organoids

**Figure 1:**
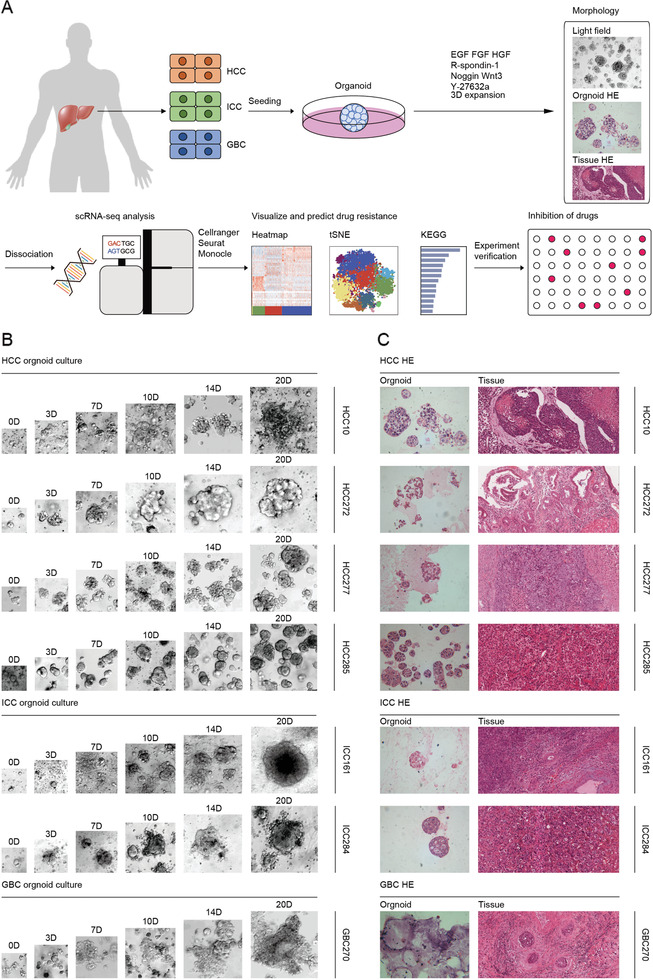
Establishment of organoids from patient‐derived hepatobiliary tumors. A) Workflow shows collection and processing of specimens of patient‐derived hepatobiliary tumor organoids for scRNA‐seq and drug screening. B) Representative bright field images of HCC, ICC, and GBC tumor organoids from seven different patients. HCC organoids form solid/compact structures, ICC tends to more irregularly shaped cyst‐like structures, and GBC organoids grow as glandular and tubular structures. Case ID was named according to histological subtypes of hepatobiliary tumor. Scale bar: 100 µm. C) Representative H&E staining of hepatobiliary tumor and derived organoid lines. Tissues generally present tumor epithelium surrounded by mesenchymal and inflammatory cells, while organoids are exclusively epithelial with tumor cell organization being remarkably well conserved. Scale bar: 200 µm. See Table 1 for detailed clinicopathological information.

To explore the cellular diversity in hepatobiliary tumors, we generated scRNA‐seq profiles using established patient‐derived hepatobiliary tumor organoids (**Figure** **1**A). Before this, we obtained hepatobiliary tumor tissue from patients who underwent surgical resection with informed consent, isolated primary tumor cells by collagenase digestion, and suspended the cells in Matrigel drops and overlaid with optimized hepatobiliary tumor organoid culture medium. To support growth and maintain long‐term expansion, a variety of small molecules and biologicals, including epidermal growth factor (EGF), fibroblast growth factors (FGF2, FGF10), hepatocyte growth factor (HGF), Wnt agonists R‐spondin1, the transforming growth factor beta (TGF‐*β*) inhibitor Noggin and the ROCK inhibitor (Y‐27632) were added (Figure 1A). In total, seven organoids from different primary hepatobiliary tumor patients were generated with detailed clinicopathological information (**Table** **1**), including four patients with hepatocellular carcinoma (HCC), two patients with intrahepatic cholangiocarcinoma (ICC), and one patient with gallbladder cancer (GBC). Individual hepatobiliary tumor organoid lines differed greatly in their morphologies as observed by bright‐field microscopy, such as solid/compact structure for HCC organoids, more irregularly shaped cyst‐like structure for ICC organoids, and glandular and tubular structure for GBC organoid (Figure 1B). Notably, after long‐term expansion in vitro, those tumor‐derived organoids maintained comparable histopathological features of their matched primary tumor tissues (Figure 1C).

**Table 1:** Clinical information of seven primary hepatobiliary tumor patients

Cell line	HCC10	HCC272	HCC277	ICC284	HCC285	ICC161	GBC270
Patient ID	206357	213567	213801	214224	214548	212495	x
Gender	M	M	M	F	F	M	M
Age	20	63	48	69	64	58	46
HBV^a)^	–	–	+	–	+	+	+
Smoking	+	–	+	–	–	+	–
Drinking	–	–	+	–	–	+	–
MVI^a)^	NA^a)^	M2	M1	NA	M0	NA	NA
Cirrhosis	–	–	+	–	–	+	–
WBC^a)^ (10^9^)	7.3	5.43	6.13	7.26	6.92	4.42	5.52
Hb^a)^ (g L^−1^)	178	137	168	125	125	151	169
PLT^a)^ (10^9^)	249	234	201	309	133	150	248
CEA^a)^ (*μ*g L^−1^)	2.5	158.9	2.1	NA	0.7	3.9	1.3
AFP^a)^ (*μ*g L^−1^)	4.4	1.4	1210	NA	168	9.9	2.6
CA724 (U mL^−1^)^a)^	0.6	211.8	NA	NA	NA	2	1.6
CA153 (U mL^−1^)	3.94	10.12	NA	NA	NA	24.4	10.65
CA199 (U mL^−1^)	9.7	5.3	18.8	NA	7.9	45.6	18.7
CA125 (U mL^−1^)	5	11	NA	NA	7.9	19.1	56.4
NSE^a)^ (ng mL^−1^)	13.73	13.59	NA	NA	NA	9.94	NA
Tumor size (mm)	NA	96*72*40	106*98	52*45*23	68*123*110	62*54	78*36

^a)^HBV, hepatitis B virus; MVI, microvascular invasion; WBC, white blood cell; Hb, hemoglobin; PLT, platelet; CEA, carcinoembryonic antigen; AFP, alpha fetoprotein; CA, carbohydrate antigen; NSE, neuron specific enolase; NA, not available.

### 2.2 Single‐Cell Analysis of Cancer Cell Signatures and Underlying Broad Drug Resistance in HCC272

**Figure 2:**
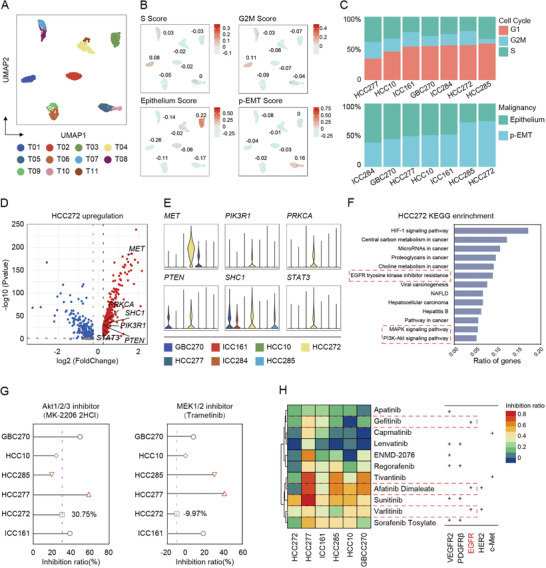
Single‐cell transcriptome atlas of patient‐derived hepatobiliary tumor organoids. A) UMAP plot of all the single cells from seven patient‐derived hepatobiliary tumor organoids reveals tumor‐specific clusters. 500 cells were extracted randomly from each sample. B) UMAP plot of all the single cells colored by different score, including S Score, G2M Score, Epithelium Score, and p‐EMT Score. Related score was determined by the average expression of representative markers genes. Color key from gray to red indicates relative score levels from low to high. For scoring gene list and scoring, see Tables S5–S7 in the Supporting Information. C) The proportions of cells with different cell cycle or malignancy of each tumor organoid. D) Volcano plots of differential expression genes of HCC272. Upregulated tumoral malignancy‐related genes were labeled. E) Violin plots showing tumoral malignancy‐related genes of each tumor organoid. The width of a violin plot indicates the kernel density of the expression values. F) KEGG enrichment analysis of HCC272 participated in a wide range of cancer‐related functions. G) Forest plot depicts inhibition ratio of MK‐2206 2HCl (Akt1/2/3 inhibitor) or Trametinib (MEK1/2 inhibitor) in six organoid lines. The assessment of each drug has been independently repeated at least twice. Data were presented as mean of multiple inhibition ratios. H) Heatmap shows inhibition ratio of 11 drugs in six organoid lines. Detailed drug information is listed in Table S3 and related inhibition ratio in Table S8 in the Supporting Information. Color key from blue to red indicates relative inhibition ratio from low to high.

Numerous studies show that single‐cell sequencing technologies offer a powerful tool to dissect intertumoral and intratumoral transcriptomic heterogeneity.^[^ ^15^, ^16^, ^17^ ^]^ To generate a delineated transcriptional landscape of tumor organoids, we established a single‐cell atlas comprising 22 505 cells collected from seven patients after initial quality controls. After normalization and principal component analysis (PCA), 500 cells randomly extracted from each sample were performed uniform manifold approximation and projection (UMAP) analysis. As with other studies, clustering of cells was primarily driven by the organoids of origin (intertumoral heterogeneity; **Figure** **2**A). Patient‐specific clustering was reflected by the unique tumor mutation characteristics of individuals (Table S1, Supporting Information). To investigate the expression pattern, we generated sample‐specific genes by performing differential gene expression analysis to identify the identity of each cell cluster (Table S2, Supporting Information). Basic biological capabilities of tumor cells were acquired during multistep development of tumors, including unlimited replicative potential and activated invasion and metastasis.^[^ ^18^ ^]^ We thus evaluated the expression of gene sets related to proliferation stages and found that HCC277 has the strongest proliferation ability with highest S phase and G2M phase score. Epithelial‐mesenchymal transition (EMT) has been widely considered as a potential driver of invasion and metastasis,^[^ ^19^ ^]^ and is increasingly recognized to be a continuous and variable process.^[^ ^20^ ^]^ Likewise, HCC272 with low expression levels of epithelial marker genes and high partial epithelial‐mesenchymal transition (p‐EMT) score, suggesting its potential tumoral malignancy (Figure 2B,C).

Given the potential tumoral malignancy, we further explored the exact gene expression pattern in HCC272. Several tumoral malignancy‐related genes (e.g., *MET*, *PIK3R1*, *PRKCA*, *SHC1*, and *STAT3*) were found highly expressed in HCC272 in comparison with other tumors (Figure 2D,E). Functional enrichment analyses showed that genes up‐regulated in HCC272 were mainly enriched for cancer‐related functions, including HIF‐1 signaling, MAPK and PI3K‐Akt signaling pathways, and EGFR tyrosine kinase inhibitor resistance (Figure 2F). To validate the role of activated signaling for cell malignance, we first subjected two inhibitors, MK‐2206 2HCl (Akt1/2/3 inhibitor) and Trametinib (MEK1/2 inhibitor), and assessed their effects on the viability of organoids. Due to the multiple activated signaling enriched in HCC272, it showed resistance to Trametinib (−9.97%) and slight sensitivity to MK‐2206 2HCl (30.75%) (Figure 2G). Since MAPK, HIF‐1, and PI3K pathways are associated with the resistance of tyrosine kinase inhibitors (TKIs), we further applied 11 TKIs (including eight drugs approved for clinical use and three in clinical trials) to examine the drug response (Table S3, Supporting Information). As shown in Figure 2H, varied drug response was pictured among different organoids, while HCC272 exhibited broad resistance to TKIs. Together, these data suggested that constitutive activation of downstream pathways such as PI3K‐Akt may be a part of the factors that tumors produce drug resistance.

### 2.3 *CD44* ^+^ Subgroup May Contribute to the Drug Resistance of HCC272

**Figure 3:**
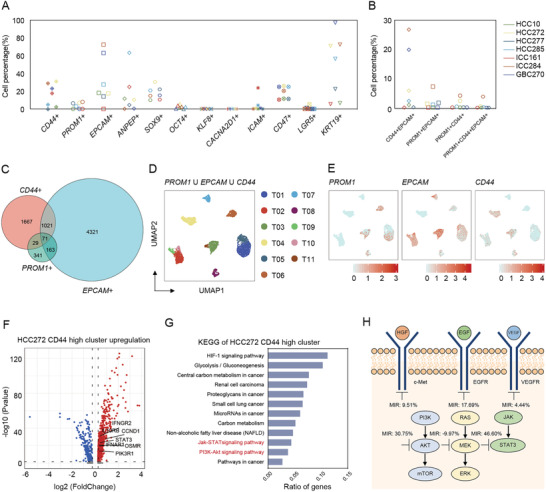
Characterizing individual organoid CSCs and its heterogeneity by single‐cell RNA‐seq. A) Scatterplots showing the cell percentage (%) of representative cell surface markers (reported as a stem marker of hepatobiliary system tumors) in individual organoids. Also see Table S4 in the Supporting Information. B) Scatterplots showing the cell percentage (%) of representative double or triple cell surface marker expressing cells in individual organoids (*PROM1*, *EPCAM*, and *CD44*). Also see Tables S9 and S10 in the Supporting Information. C) A Venn diagram is shown of single cells that expresses the representative most common hepatobiliary CSC‐like markers (*PROM1*, *EPCAM*, and *CD44*). D) UMAP plot of all the single cells marked by three hepatobiliary CSC‐like markers *PROM1*, *EPCAM*, and *CD44*. E) Expression levels of representative liver CSC‐like marker genes in each subgroup are plotted onto the UMAP map. Color key from gray to red indicates relative expression levels from low to high. The “expression level” was normalized by “logNormalize” method in “Seurat.” F) Volcano plots of differential expression genes of HCC272 *CD44* ^high^ cluster. Upregulation related genes were labeled. G) KEGG‐enrichment analysis of HCC272 *CD44* ^high^ cluster. H) Simplified scheme of the signaling pathway including target of used drugs and related inhibition ratio in HCC272 organoid line.

Tumor heterogeneity was shown to be controlled by the disruption of normal cell fate and aberrant adoption of stem cell signals.^[^ ^3^ ^]^ To better explore the cancer stemness within hepatobiliary tumor organoids, we globally examined the CSC‐like markers which was reported in previous studies. As expected, the percentage of cells with known CSC‐like markers (such as *CD133* (*PROM1*), *CD44*, *EPCAM*, *ANPEP* (*CD13*), *SOX9*, *OCT4*,^[^ ^21^ ^]^ etc.), varied greatly among individual organoid (**Figure** **3**A and Table S4, Supporting Information), of which organoid HCC272 showed a much higher percentage of CSC‐like marker positive cells. Especially, HCC272 consisted of the highest proportion of *CD44* ^+^ cells. *CD44* is involved in the tumor cell invasion and migration in liver cancer.^[^ ^22^ ^]^ To investigate the proportion of *CD44* ^+^ cells, we performed the IHC in the primary tumor tissues and identified that *CD44* ^+^ cells were significantly enriched in HCC272 (Figure S1, Supporting Information), implicating a potential role of *CD44* ^+^ cells in developing drug resistance.

UMAP analysis further revealed heterogenic distribution of three liver CSC‐like markers (*PROM1*, *EPCAM*, and *CD44*) within tumor organoids (Figure 3D,E). It should be noted that besides single positive CSCs, we observed small proportion of double positive or triple positive CSCs with distinct distribution within each organoid (Figure 3B,C). Organoid ICC284 processed the highest percentage of *PROM1*/*CD44*, *PROM1*/*EPCAM*, *CD44*/*EPCAM* double positive and *PROM1*/*CD44*/*EPCAM* triple positive cells. Interestingly, in line with the expression pattern of mono‐positive maker, higher ratio of multi‐positive (*CD44*/*PROM1*, *CD44*/*EPCAM* and *CD44*/*PROM1*/*EPCAM*) tumor cells was found in organoid HCC272 in comparison with other three HCC organoids (Figure 3B), suggesting that the abnormal enrichment of *CD44* ^+^ cells might be another responsible for its drug resistance.

To determine whether the abnormal activated pathways in *CD44* ^+^ cluster is involved in drug resistance in HCC272, we compared the difference of gene expression between *CD44* ^+^ cluster and the rest cells in HCC272 organoid. As shown in Figure 3F, total 380 genes were up‐regulated (e.g., *IFNGR2*, *IL10RB*, *CCND1*, *STAT3*, *OSMR*, *IFNAR1*, and *PIK3R1*) in *CD44* ^+^ cells. KEGG pathway enrichment analysis further revealed HIF‐1 signaling, cancer metabolism, PI3K‐Akt and Jak‐STAT pathways were activated simultaneously (Figure 3G). Jak‐STAT pathway is a principal signaling for many cytokines and growth factors and thus closely related to certain diseases, including cancer.^[^ ^23^ ^]^ The administration of STAT3 inhibitor (Cryptotanshinone) showed stronger inhibition (46.60%) on the proliferation of HCC272 than other inhibitors targeting AKT or MEK (Figure 3H), suggesting the large dependence on Jak‐STAT3 signaling in *CD44* ^+^ cells to form the drug resistance of organoid HCC272.

### 2.4 Transcriptome Signatures of Intratumoral Heterogeneity

**Figure 4:**
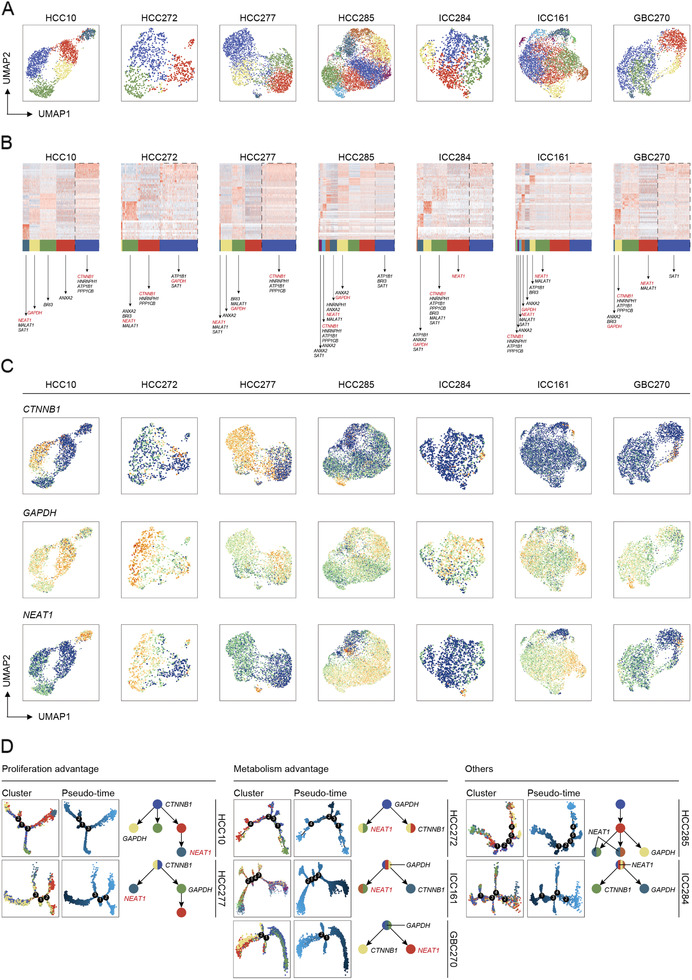
Intratumoral heterogeneity and tumor evolution trajectory of patient‐derived hepatobiliary tumor organoids. A) UMAP plot of all the single cells in individual hepatobiliary tumor organoids. Also see Table S11 in the Supporting Information. B) Heatmap shows genes (rows) that are differentially expressed in seven individual organoids clusters (columns). Ten co‐expressed genes were listed and critical genes were labeled. C) UMAP plot of all the single cells marked by *CTNNB1*, *GAPDH*, and *NEAT1* in each organoid. Color key from blue to yellow indicates relative expression levels from low to high. D) Single‐cell trajectory and pseudo‐time analysis of all the seven organoids defined the proliferation advantage cluster and the metabolism advantage one.

To better understand the intratumoral heterogeneity and their potential mechanism for cell malignance, UMAP analysis was applied to identify heterogenous clusters with diverse gene expression pattern in each organoid (**Figure** **4**A). With the same PCA ratio and resolution, organoids are divided into different number of subgroups, which indicates different level of heterogeneity within individuals. Basing on population level expression data, we screened ten co‐expressed genes (*CTNNB1 (catenin beta‐1)*, *HNRNPH1*, *ATP1B1*, *PPP1CB*, *NEAT1 (nuclear paraspeckle assembly transcript 1)*, *MALAT1*, *SAT1*, *GAPDH (glyceraldehyde‐3‐phosphate dehydrogenase)*, *ANXA2*, BRI3), of which synergetic expression of *CTNNB1*, *HNRNPH1*, and *PPP1CB* genes was found in all seven organoids with dramatically varied percentage of population (ranging from more than 50% in HCC277 to less than 5% in ICC161) (Figure 4B). Meanwhile, the distinct distribution of two genes, *GAPDH* or *NEAT1* was also observed in these organoids. *GAPDH* is a well‐known housekeeping gene, whereas recent studies reported that it could promote liver tumorigenesis by modulating glycolysis.^[^ ^24^ ^]^ *NEAT1* is a nuclear lncRNA that is an essential structural component of paraspeckles, which leads to HCC progression in response to hypoxia stress.^[^ ^25^ ^]^ UMAP analyses showed that these genes were up‐regulated with different degree in individual organoid sample (Figure 4C). Additionally, the synergetic expression of *NEAT1* and *MALAT1* was also found in the same cluster in most organoids (Figure 4B).

Importantly, trajectory and pseudo‐time analysis via Monocle further defined HCC10 and HCC277 as proliferation advantage organoids with *CTNNB1*‐enriched clusters aggregated at the beginning of evolution tree, indicating *CTNNB1* positive cluster might be the driver population for organoid proliferation. Interestingly, HCC272, ICC161, and GBC270 were designated as metabolism advantage in which *GAPDH*‐enriched cluster was found located at the top of the trunk (Figure 4D), suggesting that the reciprocal regulation of *GAPDH*‐enriched cluster was necessary for their metabolism advantage. Other two organoids, HCC285 and ICC284, showed more complicated progression trajectory. Either *CTNNB1* or *GAPDH*‐enriched cluster appeared at the later time of evolutionary process as well, whereas *NEAT1*‐enriched cluster might co‐evolved in other clusters (Figure 4D). Taken together, these data suggested that these heterogenetic cell subgroups might play the pivotal role for maintaining the development of organoids.

### 2.5 Crosstalk between *GAPDH*‐Enriched Cluster and *NEAT1*‐Enriched Cluster May Lead to the Drug Resistance of HCC272

**Figure 5:**
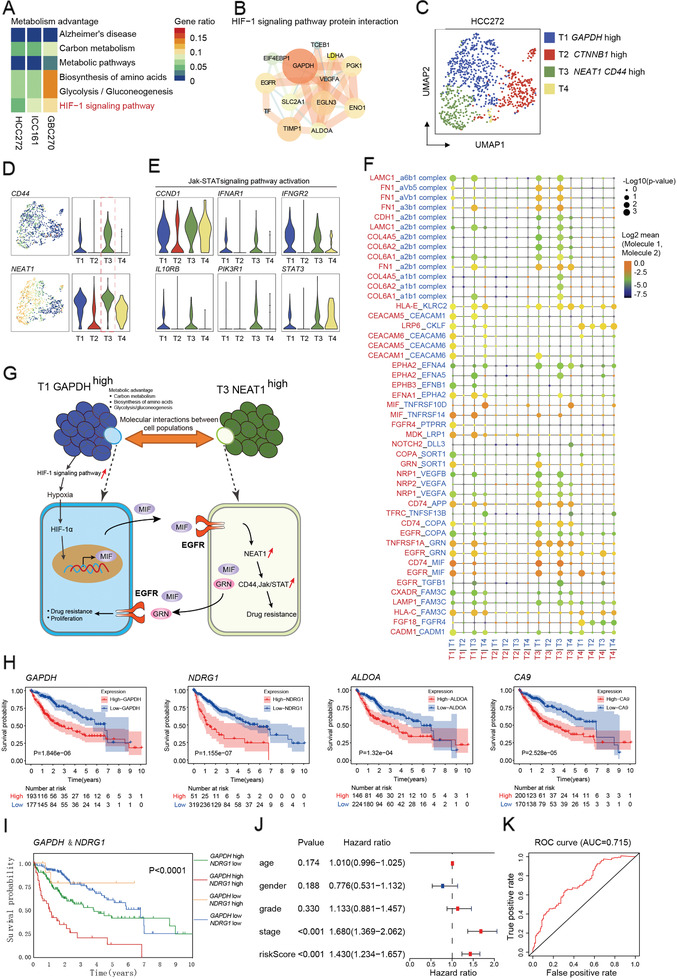
Diversified metabolic circuitry in resistant subgroups. A) Gene set enrichment analysis of metabolism advantage subpopulation in three individual organoids. B) A simplified scheme showing protein interaction in the functional interaction network of HIF‐1 signaling pathway. The interactions were generated using ingenuity pathway analysis (IPA, Ingenuity Systems). C) UMAP representation of four subgroups generated from HCC272 organoid line. D) Left: UMAP plot of HCC272 organoid line marked by *CD44* or *NEAT1*. Color key from blue to yellow indicates relative expression levels from low to high. Right: Violin plots depict corresponding gene expression of *CD44* or *NEAT1* in HCC272 organoid line. E) Violin plots of Jak‐STAT signaling pathway activation‐related genes of the subgroup in HCC272 organoid line. F) Ligand–receptor complexes specific to T1‐*GAPDH* ^high^ and T3‐*NEAT1* ^high^ clusters using CellPhoneDB. G) Overview of molecular interactions between T1‐*GAPDH* ^high^ and T3‐*NEAT1* ^high^ clusters in developing drug resistance in HCC272. H) KM plots of TCGA data divided by *GAPDH*, *NDRG1*, *ALDOA*, and *CA9* expression. I) Expression of *GAPDH* and *NDGR1* indicating different outcome in clinical. J) Forest plot of clinical indicators and riskScore (calculated by *GAPDH* and *NDGR1*). K) ROC curve of riskScore.

As *GAPDH*‐enriched cluster was identified as the primary subgroup in organoid HCC272, we wondered whether the presence of *GAPDH*‐enriched cluster might be involved in the maintenance of metabolism advantage and drug resistance. As expected, KEGG pathway analysis identified the enrichment of carbon metabolism, biosynthesis of amino acids, and glycolysis/gluconeogenesis in *GAPDH*‐enriched clusters in these three metabolism advantage organoids (**Figure** **5**A). Notably, in line with overall characteristics in organoid HCC272 (Figure 2F), HIF‐1 signaling pathway was also found enriched in *GAPDH*‐enriched cluster. Expression network analysis further revealed the central role of *GAPDH* in activating HIF‐1 signaling pathway (Figure 5B). We then inspected the distribution of *CD44* among clusters in HCC272 and found the significantly higher level of *CD44* in T3‐*NEAT1* ^high^ cluster instead of *CTNNB1* ^high^ or *GAPDH* ^high^ cluster (Figure 5C,D). Since *CD44* ^+^ cells could contribute to drug resistance by activating Jak‐STAT signaling pathway, we thus compared the expression levels of Jak‐STAT related genes in these four clusters in HCC272. As shown in Figure 5E, *CCND1*, *FNAR1*, *IFNGR2*, *IL10RB*, *PIK3R1*, and *STAT3* were significantly enriched in T3‐*NEAT1* ^high^ cluster, suggesting a regulation of *NEAT1* to *CD44* expression in T3 cluster. In the following study, we screened out another resistant organoid HCC217 by drug screening (Figure S2A, Supporting Information). It shows significant resistance to Trametinib, MK‐2206 2HCl, and 11 TKIs that we have performed to HCC272. Similarly, *CD44* ^+^ cells are highly existed in HCC217 (Figure S2B,C, Supporting Information) and particularly enriched in *NEAT1* cluster by scRNA‐seq analyses (Figure S2D, Supporting Information), in accordance with the drug resistance pattern in HCC272. Additionally, we chose two samples without resistant phenotype as controls when performing analyses, HCC25 (*CD44* low) and HCC75 (*CD44* high but without enrichment in NEAT1 cluster), implicating a potential dependence on both *NEAT1* and *CD44* within one cluster in contributing to the drug resistance. Consistent with our findings, studies have demonstrated the vital role of *NEAT1* for *CD44* expression.^[^ ^26^ ^]^

We next sought to elucidate the interaction between T1‐*GAPDH* ^high^ and T3‐*NEAT1* ^high^ clusters in developing drug resistance. CellPhoneDB analyses showed an apparently increased interactions of receptor–ligand pairs between T1‐*GAPDH* ^high^ and T3‐*NEAT1* ^high^ (Figure 5F), suggesting a close cell–cell communications among these two clusters. Particularly, *EGFR*‐*MIF* and *CD74*‐*MIF* are dominant in T3‐T1 communication (Figure 5F). MIF, upregulated by *HIF‐1α* through potential hypoxia induced mechanism,^[^ ^27^ ^]^ is the key ligand secreted from T1‐*GAPDH* ^high^ for activating *EGFR* pathway in T3 cluster. The epidermal growth factor receptor (*EGFR*) pathway plays an important role in the carcinogenesis of HCC and could mediate the expression of *NEAT1*.^[^ ^28^, ^29^ ^]^ Meanwhile, the interaction of *CD74*‐*MIF* could mediate pathologic proliferation of T3 cluster.^[^ ^30^ ^]^ Interestingly, CellPhoneDB analyses revealed the similar feedback regulation from T3‐*NEAT1* ^high^ to T1‐*GAPDH* ^high^. The *GRN* and *MIF* secreted by T3‐*NEAT1* ^high^ conferred the capacity of drug resistance and proliferation to T1‐*GAPDH* ^high^ by activating *EGFR* pathway, implicating a regulatory circuit between T1‐*GAPDH* ^high^ and T3‐*NEAT1* ^high^ in HCC272 (Figure 5G).

Since *GAPDH*‐enriched cluster with high metabolic status plays a pivotal role in shaping the hypoxia tumor environment, we examined the gene expression pattern of *GAPDH*‐enriched clusters and identified the common shared genes including *GAPDH*, *NDRG1* (N‐Myc downstream regulated 1), *ALDOA*, and *CA9* in these metabolism advantage organoids. To further demonstrate the clinical relevance of the common shared genes, we used the TCGA dataset and found a significant association between high expression of the genes and poor patient OS when compared to patients whose tumors showed a lower expression of the common genes (Figure 5H). Besides, patients with high expressions of both *GAPDH* and *NDRG1* tend to have the worst prognosis in comparison with other groups (Figure 5I). Multivariate analysis further verified the combination of *NDRG1* and *GAPDH* is an independent risk factor for disease progression (Figure 5J) with superior accuracy (AUC = 0.715) to predict clinical survival (Figure 5K). Taken together, these findings support the assertion that metabolism advantage with enrichment of *GAPDH*‐enriched cluster reshapes the hypoxia microenvironment and interplay between distinct subpopulations might enhance tumor malignant phenotypes and render worse prognosis.

## 3 Discussion

Tumor heterogeneity in hepatobiliary tumor represents a main obstacle to personalized cancer treatment. Thus, it is highly desirable to explore such heterogeneity and its impacts on drug response using a research model that can faithfully recapitulate the in vivo phenotype of hepatobiliary tumors. Single‐cell genomic provides a viable strategy to understand the genetic and phenotypic diversity at the single‐cell level, which may also help to understand complex ecosystems in tumor. Here, we applied scRNA‐seq to characterize patient‐derived hepatobiliary tumor organoids, which was currently recognized as the powerful tumor research model. We found evidence of inherent variable of transcriptional programs related to cell cycle and epithelial expression across hepatobiliary tumor organoids. Biological and transcriptomic heterogeneity of CSCs within tumor organoids were also found, which was related to chemo‐resistance. Interestingly, further analysis revealed that resistant subpopulations with unique metabolic circuitry were response to distinct molecular signatures and drug resistance. Our findings may provide a mechanistic explanation as to why some patients respond while others do not, and provide insight into the heterogeneity of hepatobiliary tumor organoids and define drug resistance associated with CSCs.

Among our key findings is the identification of biological and transcriptomic heterogeneity in patient‐derived hepatobiliary tumor organoids. Hepatobiliary tumors are characterized by a high degree of tumoral heterogeneity. Unlike other malignant tumors, such as breast cancer or lung cancer that has multiple markers of genetic mutations to determine tumor behaviors, the gene mutation spectrum of hepatobiliary tumors is wide and lacks clear characteristics, resulting extensive genetic and phenotypic variation. In this study, by generating transcriptional atlas of hepatobiliary tumor organoid in single‐cell level, we showed that different samples are inherently various in cell cycle and epithelial expression, which is consistent with their proliferation ability, potential drug‐resistance risk, and tumoral malignancy, respectively. Notably, we further identified multiple reported malignancy‐related genes (e.g., *MET*, *PIK3R1*, *PRKCA*, *PTEN*, *SHC1*, and *STAT3*) upregulated in HCC272, and demonstrated that these genes were mainly enriched for cancer‐related functions, which were associated with broad drug resistance.

Another important finding is the presence of CSC heterogeneity within tumor organoids, which tempers our understanding of drug resistance. CSC plasticity^[^ ^4^ ^]^ is a prominent cause of genetic heterogeneity in cancer, playing a vital role in tumor survival, proliferation, metastasis, and recurrence. First, cell surface markers analyses clearly showed that CSCs varied greatly among individual organoids. Second, it is of interest to note that *CD44* ^high^ cells in HCC272 exhibited a distinctive pattern, which might cause distinct transcription and more drug resistance than other tumor organoids. Third, by exploring co‐expressed genes, trajectory, and pseudo‐time analysis, we defined the *CTNNB1*‐enriched subpopulation as the proliferation advantage cluster and the *GAPDH*‐enriched as the metabolism advantage one. Specifically, the metabolism advantage organoid HCC272 could remodel tumor microenvironment through accelerating the usage of glucose, enhancing hypoxia‐induced HIF‐1 signaling, and leading to the upregulation of *NEAT1* in *CD44* ^high^ cells, which induce the hyper‐activation of Jak‐STAT signaling eventually caused drug resistance. It is suggested that understanding the distinctive metabolic circuitry in resistant subpopulations may help us characterize the CSC heterogeneity and predict therapeutic response.

A limitation of this study is that the findings are mainly based on a small number of clinical samples, and the interpretation of tumor heterogeneity is somewhat limited. However, scRNA‐seq still provides deconstructive analysis and the discovery of potential mechanisms provides credible help for precision treatment of individuals. Encouragingly, we have now established a huge hepatobiliary tumor organoid biobank based on gene variation spectrum and genetic characteristics of the Chinese population that might allow us to acquire high‐quality single cells for single‐cell transcriptome analysis. Further studies will be focused on using our established patient‐derived hepatobiliary tumor organoid biobank to perform integrative analysis from multiple‐levels, including genome, transcriptome, metabolome, and epigenome, to provide more valuable resources for clinical practice.

In summary, our study herein provides important insights into hepatobiliary tumor heterogeneity, especially the diversification of CSC distribution and the complexity of cell evolution trajectory. Meanwhile, we revealed that *CD44* positive subpopulation is responsible for drug resistance by hyper‐activating Jak‐STAT signaling pathway, which is induced by NEAT1 upregulated in hypoxia burden. Further studies with larger sample size should be warranted to better clarify the association between tumor heterogeneity and unfavorable clinical features after resection or drug treatment.

## 4 Experimental Section

##### Human Hepatobiliary Tumor Specimens

Fresh hepatobiliary resected tumors were collected with informed consent from patients who were enrolled at Eastern Hepatobiliary Surgery Hospital (Shanghai, China) without preoperative treatment. This study of human specimen collection was approved by the Ethics Committee of Eastern Hepatobiliary Surgery Hospital. Clinical information is available in Table 1. For each tumor specimen, a small fragment was snap frozen for histology and the remainder of the provided tissue was dissociated and processed for organoid culture.

##### Tumor Dissociation

Fresh resected tissue was minced, rinsed with phosphate buffered saline (PBS; Thermo Fisher Scientific), and incubated in digestion buffer on an orbital shaker at 37 °C. Incubation time of the specimen was dependent on the amount of collected tissue and ranged from 30 to 90 min, until the majority cell clusters were in suspension. The digestion buffer was composed of Dulbecco's modified Eagle medium (DMEM; GIBCO) with 4 mg mL^−1^ collagenase D (Roche), 0.1 mg mL^−1^ DNase I (Sigma), 2 × 10^−6^ m Y27632 (Sigma‐Aldrich), and 100 µg mL^−1^ Primocin (InvivoGen). After tissue digestion, DMEM media containing 10% fetal bovine serum was added to the suspension to inactivate collagenase D and cell suspension was then filtered through a 70 µm Nylon cell strainer and spun 5 min at 300–400 *g*. During processing, 10 µL of this cell suspension could be counted by Trypan Blue to determine the concentration of live cells. The pellet was washed in cold Advanced DMEM/F12 (Thermo Fisher Scientific) twice and kept cold.

##### Hepatobiliary Tumor Organoids Culture

The pellet was then resuspended with optimized hepatobiliary tumor organoid culture medium, which was composed of Advanced DMEM/F12 supplemented with 1% penicillin/streptomycin, 1% GlutaMAX‐I, 10 × 10^−3^ m HEPES, 100 µg mL^−1^ Primocin, 1:50 B27 supplement (without vitamin A), 1.25 × 10^−3^ m *N*‐acetyl‐l‐cysteine, 50 ng mL^−1^ mouse recombinant EGF, 100 ng mL^−1^ recombinant human FGF10, 1 ng mL^−1^ recombinant human FGF‐basic, 25 ng mL^−1^ recombinant human HGF, 10 × 10^−6^ m forskolin, 5 × 10^−6^ m A8301, 10 × 10^−6^ m Y27632, 10%, vol/vol Rspo‐1 conditioned medium, 30%, vol/vol Wnt3a‐conditioned medium, and 5%, vol/vol Noggin conditioned media. 5000–10 000 isolated cells mixed with cold Matrigel Basement Membrane Matrix (CORNING) and 50 µL drops of Matrigel‐cell suspension were allowed to solidify on prewarmed 24‐well suspension culture plates at 37 °C for 30 min. Upon complete gelation, 500 µL of organoid medium was added to each well and plates were transferred to humidified 37 °C/5% CO_2_ incubators at either 2% or ambient O_2_. The culture was replenished with fresh media every 3−4 days during organoid growth. Dense cultures with organoids were usually passaged with a split ratio of 1:3 every 2–3 weeks by dissociation with TrypLE Express (Gibco) and re‐seeded into new Matrigel.

##### Histological Analysis and Immunohistochemistry

Tumor tissue and organoids were fixed with 4% paraformaldehyde overnight, washed, and embedded into paraffin blocks. Sections (4–5 µm) were deparaffinized and stained with hematoxylin and eosin (H&E) for histological analysis. For Immunohistochemistry, after sections were made and hydrated, they were incubated with blocking buffer with H_2_O_2_ for 15 min and boiled with citrate (pH = 6.0). After cooling down, sections were treated with pre‐blocking buffer and incubated with primary antibodies at 4 °C overnight. Sections were incubated with secondary antibodies and 3,3'‐diaminobenzidine stained. Primary antibodies were used including CK19 (ABCAM, ab52625), AFP (Thermo Fisher Scientific, PA5‐16658), CD24 (eBioscience, 14‐0242‐85), HepPar1 (NOVUS, NBP2‐45272), Arginase (ABCAM, ab91279), CD44 (ABCAM, ab157107), PROM1 (ABCAM, ab19898), and EPCAM (ABCAM, ab71916). Immunohistochemistry was performed using the Leica BOND‐MAX (Leica Biosystems).

##### Organoid Drug Screening

Information of 13 used drugs, including drug names, targets, IC50, and source, is provided in Table S3 in the Supporting Information. 10 µL of Matrigel was dispensed into 384‐well microplates and allowed to polymerize. Cells from organoid were plated (3×10^3^ per well) and cultured in 384‐well culture plates (CORNING) for 24 h, and drugs were added to the culture medium at a final concentration of 10 × 10^−6^ m. After 4 days of drugs incubation, cell viability was assayed using CellTiter‐Glo 3D Reagent (Promega) in accordance with the manufacturer's instructions. 0.1% dimethyl sulfoxide was used as a control. When the ratio of the average level of cell viability in the presence of the drugs (*n* = 2) compared to the control (*n* = 2) was under 0.5, and the suppressive effect was considered to be significant.

##### Preparation of Single‐Cell Suspensions

Organoids were harvested and dissociated into single cells following the passaging procedure described above. Single cell was resuspended with cold PBS, and 10 µL of this cell suspension was counted by Trypan Blue to determine the concentration of live cells. Living cell rate was preferably above 90%. 30 000–50 000 cells were needed to generate scRNA‐seq.

##### scRNA‐seq

CountessII Automated Cell Counter (Thermo Fisher Scientific, USA) was used to count cells waiting to be tested and the concentration was adjusted to an ideal concentration of 1×10^6^ mL^−1^. Then, cDNA was marked by 10X GemCode Technology. Gel beads containing barcode information were first mixed with cells and enzymes. Droplets were flowed into the reservoir and were collected and then dissolved and released primer sequences for reverse transcription. cDNA was used as templates to amplify polymerase chain reaction (PCR). A sequencing library was constructed by mixing products containing barcode amplification information in each droplet. First, DNA fragments were broken into 200–300 BP fragments by Biorupter Ultrasound Fragmentation Instrument. Next, DNA library was amplified by PCR with sequencing connector P5 and sequencing primer R1. Finally, prepared samples were subjected to the 10Х single‐cell sequencing analysis platform.

##### scRNA‐seq Data Analysis

"Cell Ranger" version 2.0 was utilized to convert Illumina base call files to FASTQ files. These FASTQ files were aligned to the hg19 human reference genome and transcriptome provided by 10X Genomics. The gene versus cell count matrix from “Cell Ranger” was used for downstream analysis. The raw reads were processed using the “Cell Ranger” pipeline to obtain the unique molecular identifier (UMI). The UMI counts were transformed and normalized using the “NormalizeData” function in “SEURAT” package version 2.3.1, with the normalization method set to “logNormalize” and the scale factor set to 10 000 total UMIs per cell. Cell cycle effects were adjusted by regressing out the G2/M and S phase gene expression scores using the “ScaleData” function in “SEURAT.” PCA was performed using the highly variable genes identified by the “SEURAT” function “FindVariableGenes” with default parameters. For each individual model, the number of principal components was selected based on representing 85% of total variance. The UMAP transformation was conducted on selected principal components using the “RunUMAP” function with a default perplexity value of 30.

##### Whole‐Genome Sequencing and Somatic Mutation Calling

DNA was extracted from primary tissue and patient‐derived organoid, and libraries with an insert size of 500–600 bp were prepared according to the protocol provided by Illumina. The libraries were sequenced on an Illumina Nova6000 instrument with paired reads of 75–101 bp. WGS data were treated according to the Genome Analysis Toolkit^[^ ^31^ ^]^ best practices workflow. First, raw fastq data were treated with trimmomatic (v0.39)^[^ ^32^ ^]^ for adapter trimming and low‐quality reads filtering and then aligned to hg19 human genome reference using BWA‐mem (v0.7.15).^[^ ^33^ ^]^ Samtools (v1.4)^[^ ^34^ ^]^ was used to convert the resulting SAM files to compressed BAM files and then sort the BAM files. PCR duplicates were marked with Picard, and base quality scores were recalibrated using BaseRecalibrator tool of GATK (v4.0.9.0). Next, Mutect2^[^ ^31^ ^]^ in GATK was run to call somatic point mutations and indels from the tumor‐normal paired bam files. In addition, each normal file was conducted with tumor‐only mode of Mutect2 and then creating a panel of normal file to filter out expected artifacts and germline variations. The resulted VCF files were annotated with ANNOVAR.^[^ ^35^ ^]^ Variations with allele frequency less than 0.05 were filtered out. Cancer‐related genes were identified by COSMIC and OncoKB database.^[^ ^36^, ^37^ ^]^

##### Cell Cycle and p‐MET Scoring

First, each cell is allocated a fraction of its cycle based on the expression of its G2/M and S phase marker genes. The expression levels of these marker genes should be inversely related, and cells that do not reflect these marker genes may be in the G1 phase. The “CellCycleScoring” function was used to calculate the cell cycle score and store the S and G2/M scores in the metadata, as well as the predicted classification of each cell in the G2M, S, or G1 stage. Meanwhile, two previously reported gene sets (Table S2, Supporting Information) were used to score the epithelial differentiation status and p‐EMT (partial epithelial mesenchymal transition) status of cancer cells. Scoring was used to measure and identify degrees of malignancy in different clusters and gating cells with high malignancy to predict the prognosis of patients.

##### Trajectory and Pseudo‐Time Analysis

"Monocle," an R package designed for scRNA‐seq data, was used to identify DE genes that vary across different clusters. The mean expression level of each isoform was modeled by generalized additive models (GAMs) which relate one or more predictor variables to a response variable as

(1) $$g\left(E\left(Y\right)\right)=\beta_{0}+f_{1}\left(x_{1}\right)+f_{2}\left(x_{2}\right)+\cdot \cdot \cdot +f_{m}\left(x_{m}\right)$$

where *Y* is a response variable, and *x_i_*’s are predictor variables. The function *g* is a link function, typically the log function, and *f_i_*’s are nonparametric functions, such as cubic splines or other smoothing functions. Gene expression level across cells was modeled by a Tobit model; with some approximations, Monocle's GAM is thus

(2) $$E\left(Y\right)=s\left(\Psi_{t}\left(b_{x},s_{i}\right)\right)+\varepsilon$$

where Ψ_*t*_(*b_x_*,*s_i_*) is the assigned pseudo‐time of a cell and *s* is a cubic smoothing function with (by default) three effective degrees of freedom. *ε* is the error term that is normally distributed with a mean of zero. The DE test was performed with a *x* ^2^‐approximation of the likelihood ratio test.

##### RiskScore and Evaluation of Prognostic Indicators

The selected genes were screened using TCGA database to analyze the prognostic differences of patients. RiskScore was calculated independently from the two genes *GAPDH* and *NDRG1*, and ROC curves were plotted on the basis of these scores.

##### Statistical Analysis

All statistical analyses were performed in “R” and “GraphPad Prism” (GraphPad 7.0) software. Each in vitro experiment was independently repeated at least twice. Data were analyzed as mean ± SEM. The significance of differences between the two groups was assessed by log‐rank test. Two‐sided *p* values < 0.05 were considered statistically significant. Detailed statistical methods in this paper can be found above.

## Conflict of Interest

The authors declare no conflict of interest.

## Author Contributions

Y.Z., Z.L., Y.Z., and J.F. contributed equally to this work and developed the concept and discussed experiments; Y.Z., Z.L., and Y.Z. designed, performed, and analyzed experiments, and wrote the manuscript; J.F., X.Z., and S.W. contributed to the bioinformatic analyses; Y.Z., R.W., C.S., and X.W. processed patients’ samples and provided technical assistance; J.W., S.S., and K.W. provided technical assistance; H.W., L.C., and D.G. supervised the progress of the study and edited the manuscript.

## Supporting information

Supporting Information

Supplemental Table 1

Supplemental Table 2

## Data Availability

The raw data that support the findings of this study are available from the corresponding author (Hong‐yang Wang) upon reasonable request and through collaborative investigations. Data for figures and statistical results are provided with supplementary tables.
