# Supplementary material for: Single‐Cell Transcriptome Analysis Uncovers Intratumoral Heterogeneity and Underlying Mechanisms for Drug Resistance in Hepatobiliary Tumor Organoids
Source: Adv Sci (Weinh). 2021 Mar 23;8(11):2003897. doi: 10.1002/advs.202003897 (PMC8188185; doi:10.1002/advs.202003897)
Supplement: Supplementary file 1 — Supporting Information [file ADVS-8-2003897-s002.pdf]

© 2021 Wiley-VCH GmbH

ADVANCED SCIENCE
Open Access

Supporting Information

for *Adv. Sci.,* DOI: 10.1002/advs.202003897

Single Cell Transcriptome Analysis Uncovers Intra-tumoral Heterogeneity and Underlying Mechanisms for Drug Resistance in Hepatobiliary Tumor Organoids

Yan Zhao, Zhi-Xuan Li, Yan-Jing Zhu, Jing Fu, Xiao-Fang Zhao, Ya-Ni Zhang, Shan Wang, Jian-Min Wu, Kai-Ting Wang, Rui Wu, Cheng-Jun Sui, Si-Yun Shen, Xuan Wu, Hong-Yang Wang, Dong Gao*, and Lei Chen**

Supporting Information

Single -Cell Transcriptome Analysis Uncovers Intra-tumoral Heterogeneity and Underlying Mechanisms for Drug Resistance in Hepatobiliary Tumor Organoids

Yan Zhao[#], Zhi-Xuan Li[#], Yan-Jing Zhu[#], Jing Fu[#], Xiao-Fang Zhao, Ya-Ni Zhang, Shan Wang, Jian-Min Wu, Kai-Ting Wang, Rui Wu, Cheng-Jun Sui, Si-Yun Shen, Xuan Wu, Hong-Yang Wang*, Dong Gao*, and Lei Chen*

***Corresponding author. E-mail:**

E-mail: chenlei@smmu.edu.cn. (L.C.); E-mail: dong.gao@sibcb.ac.cn. (D.G.);

E-mail: hywangk@vip.sina.com. (H.W.).

Supplementary Figure.1

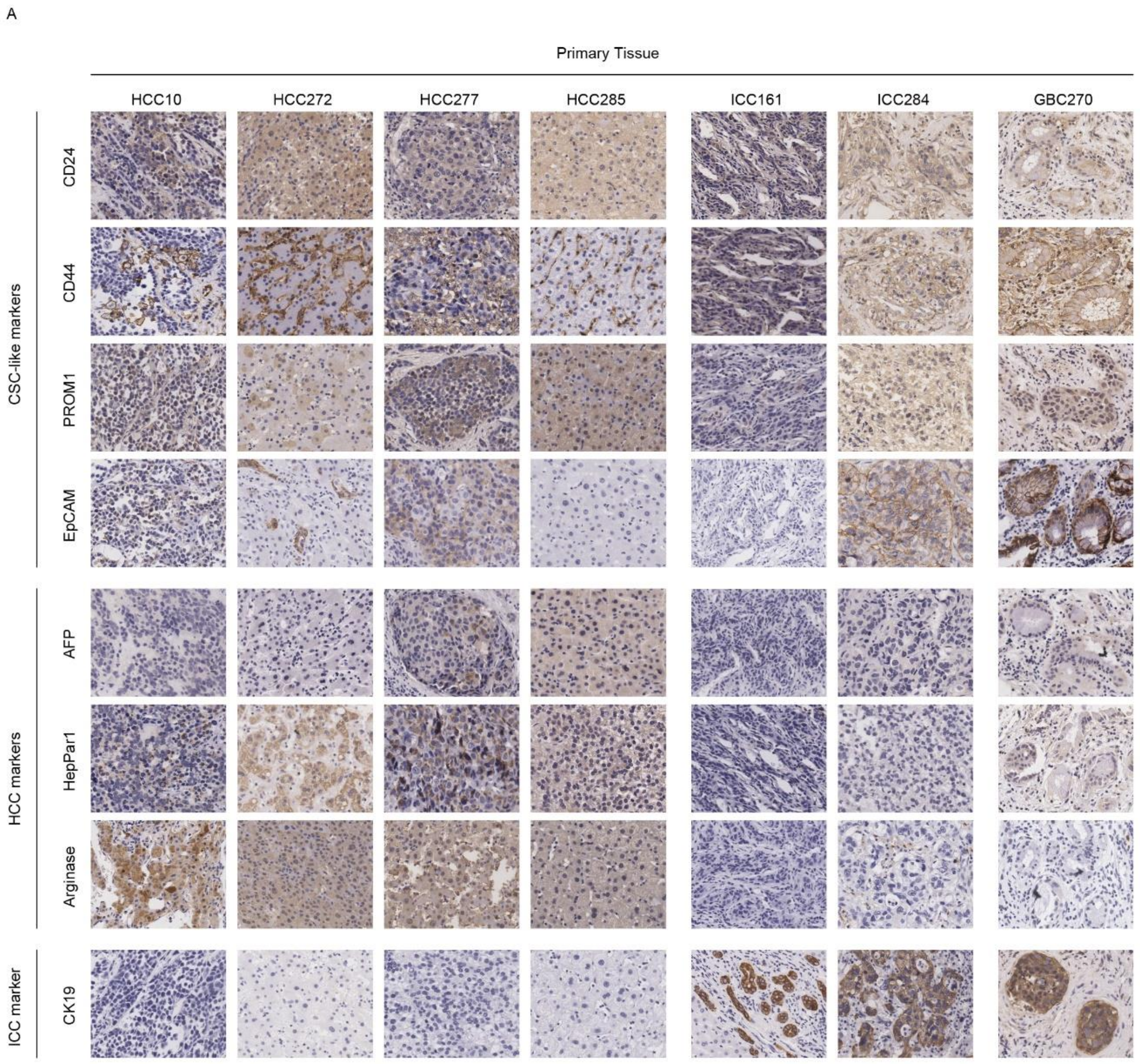

Supplementary Figure.1. Expression of CSCs-like markers and specific tumoral markers in hepatobiliary tumor tissues.

Expression of CSCs-like markers implied the CSCs heterogeneity within different hepatobiliary tumor. Particularly, *CD44*-positive cells were significantly increased in HCC272, which is consistent with analysis from scRNA-seq. In addition, diverse tumor subtypes were identified distinctly with specific expression of corresponding tumoral markers.

Supplementary Figure.2

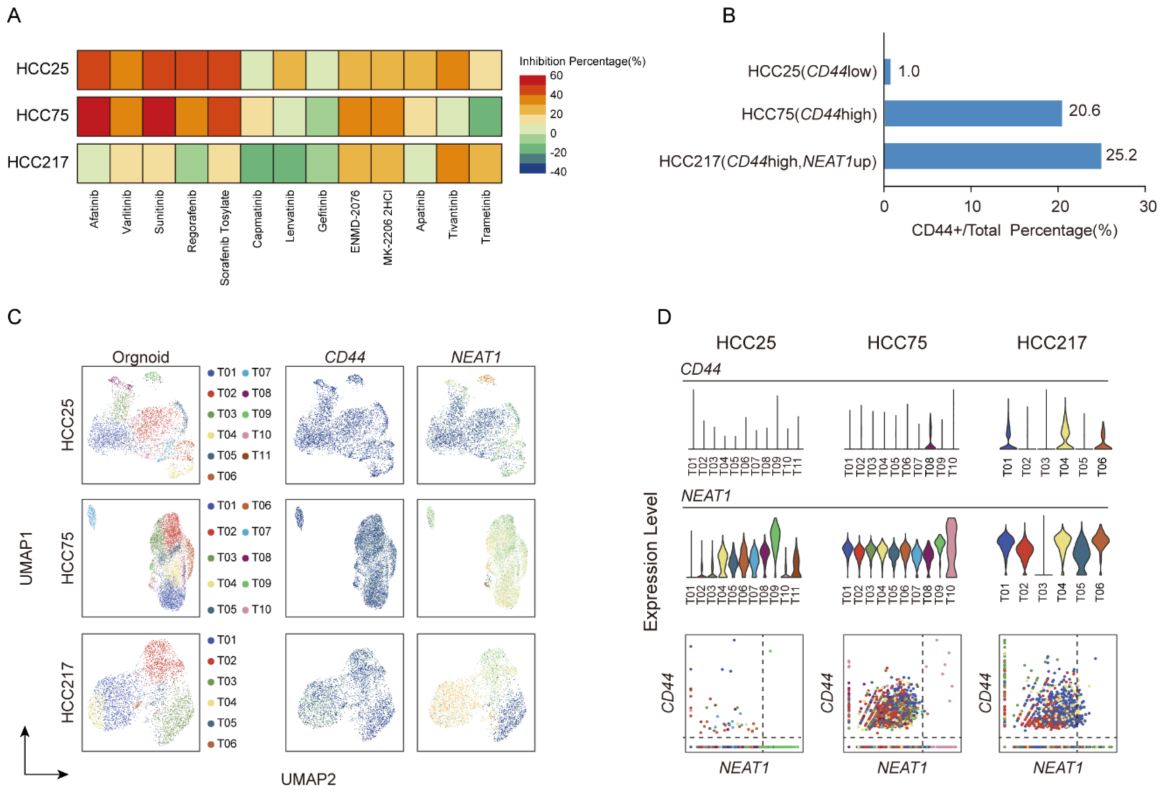

Supplementary Figure.2. ***CD44*** **and** ***NEAT1*** **common expressed in one cluster may confer to the resistant phenotype in HCC217.**

(A) Heatmap shows inhibition ratio of 13 drugs in 3 organoid lines. Detailed drug information was listed in Supplementary Table 3. Data were presented as mean of multiple independently repeated inhibition ratios.

(B) Bar plot shows the proportion of *CD44*$^+$ cells in individual organoids.

(C) UMAP plot of all the single cells in individual organoids, and cells marked by *CD44* and *NEAT1* in each organoid. Color key from blue to yellow indicates relative expression levels from low to high.

(D) Violin plots and scatterplots depict the expression of *CD44* and *NEAT1* within clusters in each organoid.
